# Anandamide inhibits Theiler's virus induced VCAM-1 in brain endothelial cells and reduces leukocyte transmigration in a model of blood brain barrier by activation of CB_1 _receptors

**DOI:** 10.1186/1742-2094-8-102

**Published:** 2011-08-18

**Authors:** Leyre Mestre, Paula M Iñigo, Miriam Mecha, Fernando G Correa, Miriam Hernangómez-Herrero, Frida Loría, Fabian Docagne, José Borrell, Carmen Guaza

**Affiliations:** 1 Neuroimmunology Group, Functional and Systems Neurobiology Department, Cajal Institute, CSIC, 28002 Madrid, Spain; 2 Department of Medical Biochemistry and Cellular Biology, Institute for Biomedicine, Sahlgrenska Academy, University of Gothenburg, Sweden; 3 INSERM, INSERM U919 'Serine Proteases and Pathophysiology of the Neurovascular Unit', GIP Cyceron, Caen Cedex, France

**Keywords:** Endocannabinoids, VCAM-1, Blood brain barrier, TMEV, Multiple Sclerosis

## Abstract

**Background:**

VCAM-1 represents one of the most important adhesion molecule involved in the transmigration of blood leukocytes across the blood-brain barrier (BBB) that is an essential step in the pathogenesis of MS. Several evidences have suggested the potential therapeutic value of cannabinoids (CBs) in the treatment of MS and their experimental models. However, the effects of endocannabinoids on VCAM-1 regulation are poorly understood. In the present study we investigated the effects of anandamide (AEA) in the regulation of VCAM-1 expression induced by Theiler's virus (TMEV) infection of brain endothelial cells using *in vitro *and *in vivo *approaches.

**Methods:**

i) *in vitro*: VCAM-1 was measured by ELISA in supernatants of brain endothelial cells infected with TMEV and subjected to AEA and/or cannabinoid receptors antagonist treatment. To evaluate the functional effect of VCAM-1 modulation we developed a blood brain barrier model based on a system of astrocytes and brain endothelial cells co-culture. ii) *in vivo*: CB_1 _receptor deficient mice (Cnr1^-/-^) infected with TMEV were treated with the AEA uptake inhibitor UCM-707 for three days. VCAM-1 expression and microglial reactivity were evaluated by immunohistochemistry.

**Results:**

Anandamide-induced inhibition of VCAM-1 expression in brain endothelial cell cultures was mediated by activation of CB_1 _receptors. The study of leukocyte transmigration confirmed the functional relevance of VCAM-1 inhibition by AEA. *In vivo *approaches also showed that the inhibition of AEA uptake reduced the expression of brain VCAM-1 in response to TMEV infection. Although a decreased expression of VCAM-1 by UCM-707 was observed in both, wild type and CB_1 _receptor deficient mice (Cnr1^-/-^), the magnitude of VCAM-1 inhibition was significantly higher in the wild type mice. Interestingly, Cnr1^-/- ^mice showed enhanced microglial reactivity and VCAM-1 expression following TMEV infection, indicating that the lack of CB_1 _receptor exacerbated neuroinflammation.

**Conclusions:**

Our results suggest that CB_1 _receptor dependent VCAM-1 inhibition is a novel mechanism for AEA-reduced leukocyte transmigration and contribute to a better understanding of the mechanisms underlying the beneficial role of endocannabinoid system in the Theiler's virus model of MS.

## Background

Vascular cell adhesion molecule-1 (VCAM-1), an endothelial receptor belonging to the immunoglobulin superfamily is a key player in leukocyte extravasation in multiple sclerosis (MS) [[1]; rev [2]]. High levels of this molecule have been found in chronic active lesions as well as in blood and CSF from MS patients [3] whereas it was hardly detectable in normal brain tissue [4]. Blockade of the interaction of VCAM-1 with its ligand, the very late antigen-4 (VLA-4), has been tested in animal models and also in clinical trials in relapsing remitting MS patients showing a significant reduction of relapse rates and MRI activity which led to the development of a new drug for MS treatment (natalizumab) [5-7]. Theiler's murine encephalomyelitis virus-induced demyelinating disease (TMEV-IDD) is a well characterized murine model of human MS, which closely resembles the chronic and progressive clinical form of the disease [8].

The endocannabinoid system (ECS), consists of endogenous ligands (AEA and 2-AG) and congeners, target receptors, synthesis (NAPE-PLD; DAG lipase), and degradation enzymes (FAAH, MAGL) and proteins involved in their transport, and intracellular trafficking [9]. Increasing evidence suggests the involvement of the ECS in both the inflammatory and the neurodegenerative processes associated to MS and other neurodegenerative diseases [rev [10,11]]. Both AEA and 2-AG possess anti-inflammatory and neuroprotective properties against harmful insults [12-16]. Controversial changes in the levels of endocannabinoids have been reported in MS and in animal models of the disease [11]. It has been suggested that the increased endocannabinoid tone might respond to an attempt to limit brain damage thus having a neuroprotective effect [13,15] whereas its decrease would be related to pathogenic processes [17]. The therapeutic potential of exogenous CBs, but also the pharmacological modulation of the ECS in animal models of multiple sclerosis has been related to their neuroprotective and anti-inflammatory activity [18-22]. A diminished number of leukocyte infiltrates into the CNS has been shown to occur in the EAE model by administering the synthetic cannabinoid WIN 5,212-2 [23]. In the TMEV-IDD model we showed that WIN 5,212-2 at the time of virus infection inhibited brain VCAM-1 expression and interfered with later disease onset [24]. However, there is still little information about the effects of endocannabinoids, and in particular of AEA, on the mechanisms involved in the control of leukocyte trafficking. Advance in the knowledge of VCAM-1 regulation by endocannabinoids may be useful to clarify the mechanisms underlying the efficacy of endocannabinoid-bases therapies. In this report, we have addressed the role of AEA in regulating 1) VCAM-1 expression in brain endothelial cells infected with TMEV and the possible receptors involved by using antagonists of the classical cannabinoid receptors, CB1 and CB2, antagonists of the vanilloid receptor TRPV1 and inhibitors of PPAR-γ receptors; 2) leukocyte transmigration in a model of BBB; and 3) in vivo brain VCAM-1 expression and microglial reactivity in TMEV-infected mice.

## Methods

### Animal and Theiler's virus inoculation

We used female Biozzi ABH and ABH mice lacking the CB_1 _receptor (*Cnr1*) gene, susceptible to TMEV-IDD development, gently gifted by Dr. Baker (University College London). Mice were maintained on food and water *ad libitum *in a 12 hours dark-light cycle. Four-to six week-old mice were inoculated intracerebrally in the right cerebral hemisphere with 10^6 ^plaque forming units (PFU) of Daniel's (DA) TMEV strain, in 30 μl of Dulbecco's modified Eagle's medium supplemented with 10% of fetal calf serum (FCS) as previously described [21,25]. Handling of animals was performed in compliance with the guidelines of animal care set by the European Union (86/609/EEC) and the Spanish regulations (BOE67/8509-12; BOE1201/2005) on the use and care of laboratory animals, and approved by the local Animal Care and Ethics Committee of the CSIC.

### Experimental procedure

At the time of TMEV infection, the mice were treated with UCM-707 (3 mg/kg, injected i.p.) twice a day (morning and afternoon) for 3 consecutive days or appropriate vehicle (5% BSA and 0.2% DMSO in phosphate-buffered saline). This dose was chosen on the basis of previous studies in our laboratory [18].

### Tissue processing and immunohistochemistry

Animal tissue was processed as previously described [24]. Briefly, mice were perfused transcardially with saline. Brains were fixed in 4% paraformaldehyde in 0.1 M PB, washed in 0.1 M PB, cryoprotected with a 7%, 15% and later 30% solution of sucrose in 0.1 M PB and frozen at -80°C until used. Free-floating coronal brain sections (30 μm thick) were processed as described previously [24] to visualize the adhesion molecule VCAM-1 (anti-VCAM-1 antibody; BD Pharmingen, San Diego, CA) and microglia (Iba-1 antibody; Wako Chemical Pure Industry, GmbH). Immunostaining was visualized with the corresponding secondary antibodies conjugated with avidin-peroxidase (Dako, Barcelona, Spain) and revealed with the choromogen 3.3' diaminobenzidine tetrahydrochloride (DAB; Sigma-Aldrich Inc, St. Louis, MO, USA) followed by counterstaining with toluidine blue. In all cases specificity of staining was confirmed by omitting the primary antibody. To quantify VCAM-1 expression fluorescence secondary antibody was used and six confocal immunofluorescence microphotograps per level were analyzed using the Image J software designed by National Institutes of Health. Results are presented as intensity of staining per vessel in case of VCAM-1 study or percentage of area occupied by CD11b^+ ^staining per field in case of microglial analysis.

### Cell cultures

*b.End5: *Murine brain endothelial cells (b.End5) which are recognized to present brain endothelium like properties were obtained from European Collection of Cell Cultures (UK). This cell model is an appropriate choice to study blood-brain barrier function [26-28]. The cells were grown in Dulbeccos's Modified Eagle's Medium supplemented with 10% heat inactivated fetal bovine serum (FBS); 1% nonessential aminoacid, 1% sodium pyruvate and 1% antibiotic penicillin and streptomycin (all from Gibco, Scotland, UK) and were maintained under standard cell culture conditions at 37°C and 5% CO_2_. One hour before experiments, cells were subjected to restricted conditions (1% FBS). In order to assess the possible receptors involved in the effects of AEA, one hour before the treatment with AEA (10 μM) and TMEV (2 × 10^5 ^pfu), cells were pre-treated with the cannabinoid receptors antagonists SR141716A (CB_1_, 1 μM); AM630 (CB_2_, 1 μM); capsazepine (TRPV1, 10 μM) or GW9662 (PPARγ, 100 nM, 1 μM).

*Astrocytes*: cell cultures were obtained as previously described [29]. Forebrains were dissociated mechanically, filtered through a 150 μm nylon mesh, resuspended in DMEM containing 10% heat-inactivated FCS, 10% heat-inactivated FBS and 1% penicillin/streptomycin and plated on poly-L-lysin-coated (5 μg/ml) 75 cm^2 ^flasks (Nunc, Wiesbaden, Germany). After 7 days in culture the flasks were shaken at 260 rpm at 37°C overnight to remove microglia and oligodendrocytes.

*Leukocytes*: Lymphatic nodes were homogenized in cold PBS with the plunger of a syringe, filtered through a 70 μm cell strainer to obtain a single cell suspension, centrifuged for 5 min at 1200 rpm and resuspended in RPMI supplemented with 10 mM HEPES (pH 7.4), 2 mM glutamine and 10% FCS, β-mercaptoethanol (50 μM).

### Adhesion assay

Confluent brain endothelial cell monolayer infected with TMEV (2 × 10^5 ^pfu) and treated with AEA (10 μM) was subjected or not to the cannabinoid receptors antagonist by pre-treatment for 1 hour with the CB_1 _or CB_2 _selective receptor antagonist, SR141716A (SR1, 1 μM) or AM630 (1 μM), respectively. After 6 hours, 2.5 × 10^5 ^leukocytes stained with calcein acetoxymethyl ester (AM) (5 μM) (Sigma-Aldrich Inc, St. Louis, MO, USA) were allowed to adhere to endothelial monolayer for 20 hours. Lapsed this time non bound leukocytes were removed, five microphotographs/field, fluorescence and phase contrast, were used for counting adhered leukocytes by Metamorph software. The assay was performed in triplicate for each value and was repeated 3 times. Calcein acetoxymethyl ester is a vital dye what is membrane permeable but becomes membrane impermeable and fluorescent when cleaved by intracellular sterases.

### Blood brain barrier model

Blood brain barrier model was performed as described previously [30] with modifications. Briefly, transwell filters (surface area 6.4 mm; pore size, 8 μm; BD Falcon™ Cell Culture Inserts) were coated with colagen type I (50 μg/ml; BD Falcon) and fibronectine (50 μg/ml; Invitrogen, Barcelona, Spain). Astrocytes (5 × 10^4 ^cell/well) were allowed to adhere to the bottom of the filter for 10 minutes in DMEM with 10% FBS, 10% FCS and 1% penicillin/streptomycin. Contamination of adherent astrocytes on the bottom of the well was avoided. After 24 hours, brain endothelial cells (b.End5) were seeded on the top of the filter at a density of 5 × 10^4 ^cell/well in DMEM with 10% FBS, 1% non-essential aminoacid, 1% sodium pyruvate and 1% penicillin/streptomycin. We consider that BBB was established when transendothelial electrical resistance was close to 200Ω/cm^2 ^[30]. Once confluent, endothelial cells were infected with TMEV (2 × 10^5 ^pfu) and treated with AEA (10 μM). To study the involvement of cannabinoid receptor, cells were pre-treated with the CB_1 _or CB_2 _receptors antagonist, SR1 (1 μM) or AM630 (1 μM) respectively. Following stimulation for 6 hours, 2.5 × 10^5 ^leukocytes were added on the top of the insert for 20 hours. The entire transmigrating cell populations present in the bottom chamber were collected and counted by using a hemocytometer. For schematic illustration of the BBB model see additional file 1A and additional file 2.

### Permeability assay

Permeability assay was performed as described [31]. Briefly, after rinsed in phenol-red-free DMEM the top and the bottom of the filter, 400 μl of 10% FBS/phenol-red-free DMEM and 200 μl of 0.45% albumin conjugated to Evan's blue dye were added to the bottom and the top of the well, respectively and incubated at 37°C for 30 min. Absorbance of the bottom medium was read at 620 nm [see Additional file 1B].

### Immunocytochemistry

To visualize the tight junction zonula occludens-1 (ZO-1) in the blood brain barrier model, cells were fixed with 4% paraformaldehyde, washed with PBS and incubated overnight at 4°C with the primary antibody (ZO-1, Zymed Laboratorioes, Carlsbad, CA) in PBS containing 5% NGS and 0,1 Triton X-100. After washing with PBS, cells were incubated for 1 h at RT with secondary anti-rabbit antibody IgGs, conjugated with Alexa 488 (Molecular Probes, Eugene, OR, USA) washed with PBS and mounted on glass slides with fluorescent mounting medium. In all cases, specificity of staining was confirmed by omitting the primary antibody [see Additional file 1C].

### ELISA

Soluble fraction VCAM-1 (sVCAM-1) content in endothelial cells supernatants was measured by solid phase sandwich ELISA, using a monoclonal antibody specific for mouse sVCAM-1 (R & D Systems Inc., MN, USA), according to the manufacturer's instructions. The assay sensitivity was 30 pg/ml.

### Statistical analysis

All results are presented as mean ± SEM. For in vitro experiments the n value corresponds at least to three independent experiments; with triplicate determinations in each experiment. One-way ANOVA, followed by a *post hoc *Tukey's multiple comparison tests was used to examine the statistical significance of *in vitro *assays. Repeated measure test and post hoc Duncan test was used to analyze the statistical significance of VCAM-1 and CD11b studies. *p *values < 0.05 were considered significant.

## Results

### Anandamide inhibits VCAM-1 induced by TMEV in brain endothelial cells by CB1 receptors

**Figure 1:**
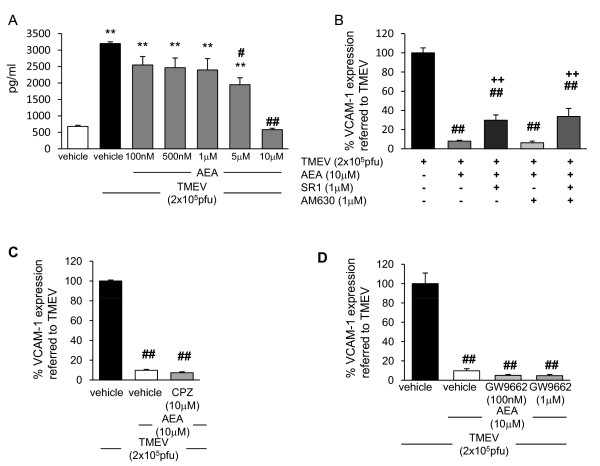
**Anandamide inhibits VCAM-1 production induced by TMEV through a mechanism that involves CB_1 _receptor**. (**A**) sVCAM-1 levels were measured by ELISA in supernatants of cell cultures 20 h after AEA treatment (100 nM, 500 nM, 1 μM, 5 μM, 10 μM). Confluent TMEV-infected brain endothelial cell monolayers were pretreated for 1 hour before AEA treatment with (**B**) the cannabinoid receptor antagonist SR1 (1 μM) or AM630 (1 μM); (**C**) the vanilloid receptor antagonist capsazepine (10 μM); (**D**) the PPARγ receptor antagonist GW9662 (100 nM and 1 μM). Results show the means ± SEM from three independent experiments done in triplicate. (**p < 0,01 vs. vehicle; ##p < 0.01 vs. TMEV+vehicle; ++p < 0.01 vs. TMEV+AEA, ANOVA followed by Tuckey's test).

The endothelial blood brain barrier protects the CNS from the changing environment in both physiologic and pathologic conditions. Previous work in our lab has demonstrated that sVCAM-1 is constitutively expressed on b.End5 cells and increased by TMEV infection [24]. We first analyzed the effect of AEA on the production of VCAM-1 by TMEV-infected brain endothelial cells. Dose response studies of AEA on sVCAM-1 production showed that 10 μM was the most effective dose to prevent the expression of VCAM-1 induced by TMEV at 20 hours postinfection (Figure 1A). AEA also inhibited VCAM-1 expression in resting cells (data not shown). Down-regulation of VCAM-1 induced by AEA (10 μM) was partially reversed by the addition of the CB_1 _receptor antagonist, SR141716A (SR1) but not by the CB_2 _receptor antagonist AM630 (Figure 1B). The doses used for CB antagonists were 1 μM on the basis of their capability for antagonizing CB effects in our previous work. To examine if vanilloid receptors expressed in brain endothelial cells [32] were involved in AEA inhibition of VCAM-1 we pretreated the cells with capsazepine (10 μM). As shown in Figure 1C, the blockade of vanilloid receptors did not modify the inhibitory effect of AEA on VCAM-1 expression. In addition we explored the role of PPARγ receptors as it has been described to mediate some of the actions of AEA [reviewed by [33]]. In our study the treatment with the inhibitor of PPARγ, GW9662 (at nanomolar and micromolar doses) did not prevent AEA-induced VCAM-1 inhibition (Figure 1D). In conclusion, AEA-induced inhibition of VCAM-1 in brain endothelial cells implies the activation of CB_1 _receptors.

### Anandamide limits leukocyte migration through a blood brain barrier model by a mechanism involving CB1 receptors

**Figure 2:**
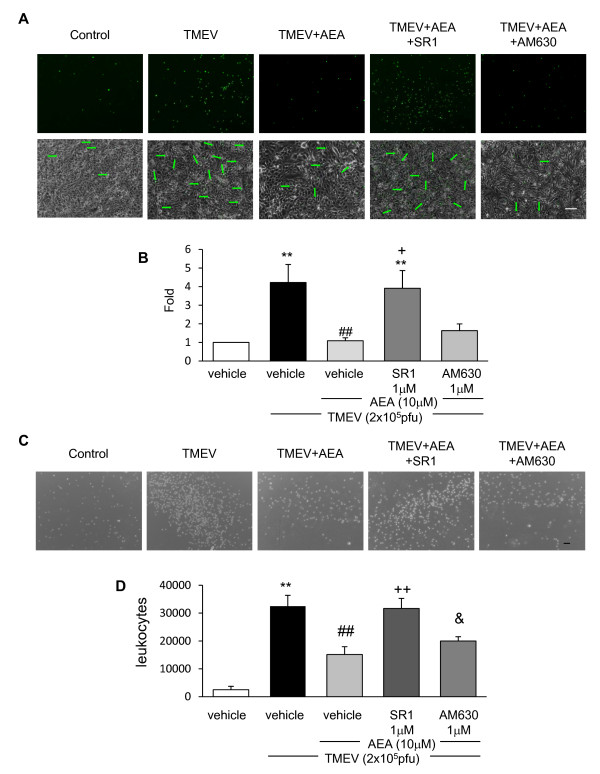
**AEA limits leukocyte adhesion to TMEV stimulated brain endothelial cells and leukocyte transmigration through *in vitro *BBB by CB_1 _involvement**. Brain endothelial cell monolayer were stimulated with a combination of TMEV (2 × 10^5 ^pfu), AEA (10 μM), SR1 (1 μM) or AM630 (1 μM) for 6 hours. After that, 2.5 × 10^5 ^leukocytes stained with AM-calcein (5 μM) were added to the endothelial culture for 20 hours. (**A**) Representative immunofluorescence microphotographs of the leukocytes stained with AM-calcein adhered to the brain endothelial cell monolayer in each case and phase contrast microphotographs of brain endothelial monolayer merged with immunofluorescence microphotographs of AM-calcein stained leukocytes bring out with arrows. Scale bar 100 μm. (**B**) Quantification of leukocytes adhered to brain endothelial monolayer in each case normalized to control group (n = 6). (**p < 0.01 vs. vehicle; ##p < 0.01 vs. TMEV; +p < 0.05 vs. TMEV+AEA, ANOVA followed by Tuckey's tests). (**C**) TMEV (2 × 10^5 ^pfu), plus AEA (10 μM), or plus SR1 (1 μM) or AM630 (1 μM) were added to the upper side of the insert (endothelial culture) and IL1-β (10 ng/ml) was added to the bottom side (astrocyte culture) for 6 hours. 2.5 × 10^5 ^leukocytes were added to the upper side of the insert for 20 hours and representative phase contrast microphotographs of leukocytes crossed to bottom side of the insert were taken. (**D**) Quantification of leukocytes in the bottom side of the insert after 20 hours of experiment. (**p < 0.01 vs. vehicle; ##p < 0.01 vs. TMEV+vehicle; ++p < 0.01 vs. TMEV+AEA; &p < 0.05 vs. TMEV+AEA+SR1, ANOVA followed by Tuckey's test; n = 6).

VCAM-1 is critically involved in leukocyte transmigration into the CNS. Therefore, our next step was to assess the functional relevance of AEA-induced VCAM-1 inhibition in leukocyte transmigration. First, we showed that leukocyte adhesion to TMEV-infected endothelium was significantly increased (p < 0.01) in comparison to cell adhering to resting cell monolayer. Importantly, the treatment with AEA (10 μM), at the time of virus infection diminished leukocyte adhesion (p < 0.01) (Figure 2A). In agreement with the involvement of CB_1 _receptors in AEA-induced VCAM-1 inhibition, the pretreatment with the CB_1 _receptor antagonist (SR1), but not with the CB_2 _antagonist (AM630), reversed the inhibitory effect of AEA on leukocyte adhesion (Figure 2A). Quantification is presented as a ratio of number of leukocyte adhered to the endothelial cell monolayer in each group normalized to control group (Figure 2B).

Next, we analyzed whether the effect of AEA on the adhesion of leukocytes interferes on leukocyte transmigration through the BBB model. As expected TMEV-infection of brain endothelial cells increased the number of leukocytes crossed the BBB model referred to control (Figure 2C). Accordingly to our results in the experiments of leukocytes adhesion the treatment of endothelial cells with AEA (10 μM) diminished leukocyte crossing by a mechanism that involves CB_1 _receptors. Figure 2D shows the quantification data on the number of leukocytes that cross the BBB model.

### The increased anandamide tone inhibits VCAM-1 expression in Theiler's virus-infected mice

**Figure 3:**
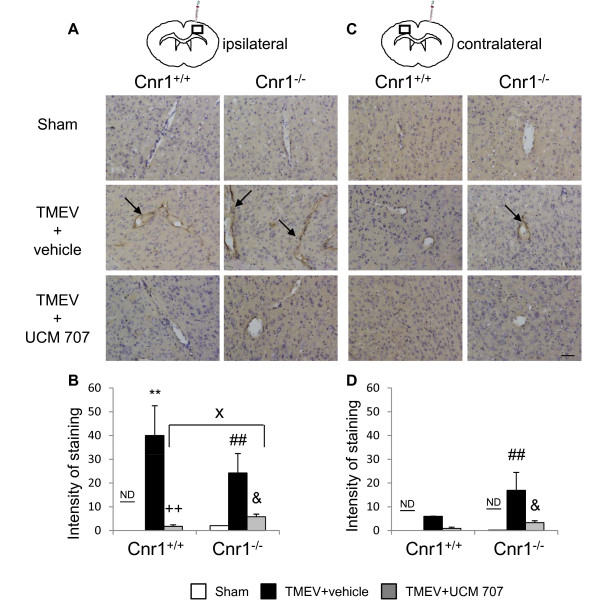
**The treatment with UCM-707 inhibits VCAM-1 expression in TMEV-infected mice**. Study with Cnr1^+/+ ^and Cnr1^-/- ^mice. Both, TMEV-infected and Sham mice were treated with UCM-707 (3 mg/kg) or the corresponding vehicle (n = 3 for each group) immediately after virus infection for three consecutive days. Analysis were performed using representative microphotographs of coronal brain sections (30 μm) of ipsilateral (**A**) or contralateral (**C**) brain tissue close to the virus side of injection, immunostained for VCAM-1. Arrows indicate VCAM-1 immunostaining. Scale bar is 50 μm. (**B, D**) Quantification of intensity of VCAM-1 staining as described in Material and methods in the ipsilateral or contralateral hemispheres, respectively. ND, non detected; **p < 0.01 vs. Sham (Cnr1^+/+^); ##p < 0.01 vs. Sham (Cnr1^-/-^); ++p < 0.01 vs. TMEV+vehicle (Cnr1^+/+^); &p < 0.05 vs. TMEV+vehicle (Cnr1^-/-^); Xp < 0.05 vs. TMEV+UCM-707 (Cnr1^+/+^).

On the basis of our *in vitro *results, we next analyze the effect of the pharmacological modulation of the AEA tone on VCAM-1 response against TMEV infection in vivo, using wild type and CB_1 _knockout mice (Cnr1^-/-^). Accordingly to other studies [24], VCAM-1 expression was not detected, by immunohistochemistry, in the brains of sham animals in both type of mice, Cnr1^+/+ ^or Cnr1^-/-^. The intracranial injection of TMEV induced the expression of VCAM-1 in the ipsilateral cerebral cortex surrounding blood vessels close to the site of injection in both type of mice, Cnr1^+/+ ^as well as Cnr1^-/- ^mice (Figure 3A). Corroborating our in vitro findings, the treatment with the inhibitor of AEA uptake UCM-707 induced a significant reduction of VCAM-1 expression in TMEV-infected mice (Figure 3A). Although, UCM-707 decreased VCAM-1 expression in Cnr1^+/+ ^and Cnr1^-/- ^mice, quantification analysis (Figure 3B) revealed that the degree of VCAM-1 reduction in the ipsilateral cerebral cortex of Cnr1^+/+ ^mice was significantly higher than that observed in Cnr1^-/- ^(p < 0.05). This observation suggests the participation of CB_1 _receptors in the effects of UCM-707 treatment. Additionally, when we analysed the contralateral hemisphere (Figure 3C) we found that only mice lacking CB_1 _receptors showed increased VCAM-1 expression in the vasculature in response to TMEV that was significantly inhibited by the treatment with UCM-707 as revealed the quantification of staining intensity (Figure 3D).

### The increased anandamide tone limits microglial activation in mice infected with Theiler's virus

**Figure 4:**
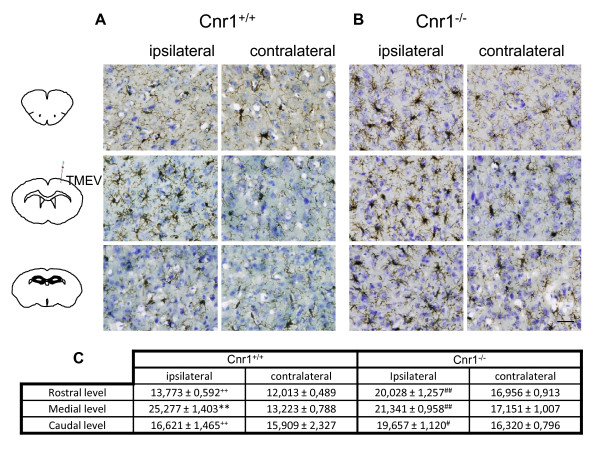
**CB_1 _deletion exacerbates microglial response against TMEV infection**. Coronal brain sections (30 μm) were obtained from Cnr1^+/+ ^TMEV-infected mice (**A**) or Cnr1^-/- ^TMEV-infected mice (**B**), stained for CD11b with Iba-1 antibody and counterstained with toluidine blue (n = 3 for each group). To perform the analysis of microglia phenotype morphology brain tissue was studied in both hemispheres and at rostral, medial and caudal levels. (**C**) Quantification of percentage of area occupied by microglia per field is represented. Scale bar is 50 μm. **p < 0.01 vs. contralateral (Cnr1^+/+^); #p < 0.05 vs. contralateral (Cnr1^-/-^); ##p < 0.01 vs. contralateral (Cnr1^-/-^); ++p < 0.01 vs. medial level (Cnr1^+/+^).

The intracranial injection of Theiler's virus induced an increase of microglia with reactive morphology in the cerebral cortex at the level of infection (medium level) but only in the ipsilateral infected hemisphere (Figure 4A). Interestingly, the microglial response was exacerbated in Cnr1^-/- ^mice (Figure 4B), now extending from prefrontal cortex (rostral level) to hippocampal level (caudal level). When we analyzed the contralateral hemisphere we found that microglial cells did not show reactive morphology at the three brain levels examined in the wild type mice as well as in Cnr1^-/- ^mice. Therefore, in response to TMEV infection, activation of microglial cells only occurred in the ipsilateral hemisphere. Quantification analysis of percentage of area occupied by microglia per field was summarized in Figure 4C.

**Figure 5:**
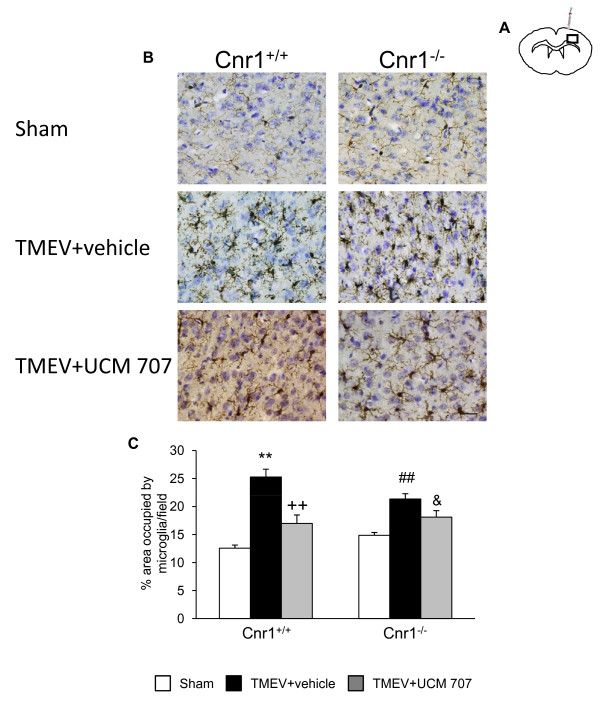
**The treatment with UCM-707 decreases microglia reactivity in TMEV-infected mice**. Study with Cnr1^+/+ ^and Cnr1^-/- ^mice. Both TMEV-infected and Sham mice from both strains (Cnr1^+/+ ^and Cnr1^-/-^) were treated with UCM-707 (3 mg/kg) or the corresponding vehicle (n = 3 for each group) immediately after the virus infection for three consecutive days. (**A**) Coronal brain section level for the analysis of CD11b^+ ^expression. (**B**) Representative micrographs of ipsilateral cerebral cortex in sham, TMEV-infected plus vehicle or TMEV infected plus UCM-707 from Cnr1^+/+ ^or Cnr1^-/- ^mice. (**C**) Quantification of percentage of area occupied by microglia per field is represented. Scale bar is 50 μm. **p < 0.01 vs Sham (Cnr1^+/+^); ++p < 0.01 vs. TMEV+vehicle (Cnr1^+/+^); ##p < 0.01 vs Sham (Cnr1^-/-^); &p = 0.07 vs. TMEV+vehicle (Cnr1^-/-^).

The treatment with UCM-707 significantly (p < 0.01) reduced the presence of microglia with reactive morphology in Cnr1^+/+ ^mice (Figure 5B) at the medium level close to the site of injection (Figure 5A). Cerebral cortex sections from Cnr1^-/- ^mice showed a tendency toward diminishing microglia reactivity but without reaching statistical significance (p = 0,07; Figure 5C). The analysis of the contralateral hemispheres didn't reveal the presence of microglia with reactive morphology (data not shown).

## Discussion

The ECS has been suggested to contribute to the maintenance of homeostasis between the immune and the nervous systems [34,35]. Besides, the pharmacological activation of ECS is emerging as a potential therapeutic strategy for neurodegenerative diseases including multiple sclerosis [rev [10,36]]. Mechanisms underlying the beneficial effects of CBs on MS are not fully clarified; however, anti-inflammatory and/or neuroprotective actions seem to be involved [37]. Leukocyte migration into the CNS is widely recognized as a pivotal event in the development of MS in which adhesion molecules like VCAM-1 are critically involved and emerge as marker of endothelial activity [rev [2,7]].

The notion that restriction of immune cells traffic into the CNS by CBs could represent a novel mechanism to suppress brain immune reactivity was first suggested by two laboratories in both, TMEV-IDD and EAE models by using the synthetic agonist WIN 55,212-2 [21,23]. In the present study we show that the endocannabinoid, AEA inhibits the expression of VCAM-1 in TMEV-infected brain endothelial cells resulting in reduced leukocyte adhesion and crossing through an *in vitro *model of BBB. In the TMEV-IDD model, cumulative evidence suggests that TMEV may enter the CNS by infection of cerebrovascular endothelial cells. Thus, infection of endothelial cells might represent one of the first events in the pathogenesis of TMEV-induced demyelination. The persistence of TMEV in cloned mouse cerebrovascular endothelial cells appears to support this concept [38]. Pioneering studies on TMEV-IDD showed that adhesion molecules play a critical role in leukocyte extravasation [39] pointing out the interest of a reduction of VCAM-1 expression by AEA. CB_1 _and CB_2 _receptors were expressed in b.End5 as well as in primary cultures of murine brain endothelial cells [40]. Most of the effects of CBs are mediated by their specific receptors CB_1 _and CB_2 _that are asymmetrically distributed in the BBB. CB_1 _receptor is mainly located at the luminal side while CB_2 _receptors are on the abluminal side of the endothelium [41,19]. In our study, VCAM-1 suppression by AEA in brain endothelial cells was mainly mediated by the activation of CB_1 _receptors. Most importantly, AEA-induced inhibition of leukocyte adhesion and crossing through the BBB also involved CB_1 _receptors accordingly to the specific distribution of this type of receptors in the BBB. In agreement with our observations, studies on HIV-1 Gp120-effects in brain microvascular endothelial cells have shown that CB_1 _based synthetic CBs prevented monocyte transmigration across a human model of BBB [42]. Although CB_1_, CB_2 _[40] and TRPV1 [32] receptors are expressed in murine brain endothelial cells, our results ruled out the involvement of CB_2 _and TRPV1 receptors in AEA-induced inhibition of VCAM-1. Differential expression of CB_2 _receptors and NAPE-PLD (the major enzyme associated with synthesis of AEA) in cerebral endothelium at different stages of MS has been recently reported [43]. In the above study, increased CB_2 _receptor staining was associated with BBB disruption in active plaques from MS tissue samples, suggesting a role for endothelial CB_2 _in the protection and/or repair of BBB injury. However, previous studies of MS brain tissue did not find endothelial expression of CB_2 _[44,45]. In TMEV-infected brain endothelial cells the possibility that AEA activates PPAR-γ receptors [33] to suppress VCAM-1 can be also discharged despite the fact that PPARs agonists prevent the interaction of leukocytes with stimulated endothelium [46].

The majority of studies on AEA actions in endothelial cells have focused on its vasodilator and hypotensive activity and there were discrepancies on the type of receptor implicated, probably due to differences between peripheral and brain endothelial cells [47]. Using mouse cerebral endothelial cells and consistent with our results, AEA-induced increased COX-2 expression involves the activation of CB_1 _receptors [48].

Although alterations in the ECS during the course of MS have been suggested to represent a protective physiological strategy [13,18,49,50] the role of endocannabinoids in MS remains uncertain. While most of studies on ECS and MS focused on established disease, understanding the role of endocannabinoids during the induction phase would be an important point as exacerbated leukocyte trafficking into the CNS represents a key stage in the disease. Therefore, here, we investigated the role of CB_1 _receptors and the effects of the inhibitor of AEA uptake, UCM-707, on VCAM-1 expression in wild type and CB_1 _knockout mice (Cnr1^-/-^) during the early phases of TMEV-IDD. Intracranial infection with TMEV induced the expression of VCAM-1 in surrounding blood vessels close to the site of injection in Cnr1^+/+ ^as well as in Cnr1^-/- ^mice whereas VCAM-1 was not detected in brains of sham animal in both type of mice accordingly to other studies [4,24]. The treatment with UCM-707 resulted in down-regulation of VCAM-1 expression in both type of mice. However, the degree of inhibition of VCAM-1 in the ipsilateral cerebral cortex of Cnr1^+/+ ^mice was significantly higher than that observed in Cnr1^-/- ^mice supporting the involvement of CB_1 _receptors and corroborating our *in vitro *results. In addition, the analysis of the contralateral hemisphere showed increased VCAM-1 expression only in the vasculature of Cnr1^-/- ^mice that was inhibited by UCM-707. Thus, our *in vivo *data confirm the importance of CB1 receptors but, suggest that besides CB1 receptors, additional mechanisms are contributing to the effects of UCM-707 on VCAM-1 inhibition. It is difficult to have the overall picture of what is happening as consequence of increasing AEA tone under the conditions of our study due to the multiple cellular targets for AEA actions on the responses to TMEV infection. Nevertheless, we have shown here that AEA by targeting brain endothelial cells may interfere with leukocyte recruitment across the BBB through the inhibition of VCAM-1.

As suggested in the cardiovascular endothelium [51,52] in the brain endothelium AEA and other endocannabinoids like 2-AG, would be synthetized and released from a nearby source such as astrocytes [53], microglia [54] and even from the own endothelial cells to regulate the response of brain endothelium to different stimuli as we observed in the case of TMEV. The observation that NAPE-PLD expression is elevated on blood vessels and in reactive astrocytes distributed closely around them suggests the synthesis of AEA by brain endothelium in MS [43]. In other models of brain injury 2-AG has been shown to be released and to reduce BBB damage [14,55].

Additionally, endocannabinoids may control brain innate immunity in MS by acting in different CNS cell types such as astrocytes and microglial besides immune cells [rev [56]]. Activating or inhibiting the innate immune response influences the development of TMEV-IDD [57]. In this line, AEA enhances IL-6 production in astrocytes infected with TMEV by a CB_1 _receptor-mediated pathway [58] and in a more recent work AEA modulates TMEV-induced IL-12, IL-23 and IL-10 in microglia by activating CB_2 _receptors [59].

An important finding of the present study is that the lack of CB_1 _receptor leads to an exacerbation of microglial response to TMEV infection in the ipsilateral hemisphere. Thus, microglial activation was observed from prefrontal cortex to hippocampal levels instead of maintaining it exclusively in the area close to the injection site. Currently, we unknown the meaning of the extensive microglial activation in Cnr1^-/- ^mice, but it is likely to be associated with the facilitation of spreading viral antigens as microglia/macrophages are an important virus reservoir [60]. In line with a protective role of CB_1 _receptors previous studies have reported that CB_1_-knockout mice develop more severe CREAE [61,62]. Moreover, recent studies reveal that repeat polymorphism of the *Cnr1 *gene could represent a genetic risk factor for both the primary progressive [63] and relapsing-remitting form of MS [64].

## Conclusions

In summary, mechanisms underlying the decreased cellular infiltration on the CNS by CBs in animal models of MS are not yet clear but the present study showed that anandamide was effective in reducing endothelial VCAM-1 expression and BBB permeability via CB_1 _receptors. More relevant is that the inhibitor of anandamide uptake, UCM-707 reduced VCAM expression in TMEV infected mice with the participation of CB_1 _receptors. Available data from MS patients subjected to success natalizumab therapy showed downregulation of sVCAM-1 which is considered a good biomarker of endothelial activity [7]. The inhibition of VCAM-1 expression in cerebral vasculature by anandamide provides a new mechanism that may explain the therapeutic action of increased anandamide tone in neuroinflammatory diseases like MS.

## List of abbreviations

2-AG: 2-Arachidonoylglycerol; AEA: N-arachidonoylethanolamine or anandamide; BBB: blood brain barrier; CBs: cannabinoids; CNS: central nervous system; CREAE: chronic experimental autoimmune encephalomyelitis; DAG lipase: Diacylglycerol lipase; EAE: experimental autoimmune encephalomyelitis; ECS: endocannabinoid system; FAAH: Fatty acid amide hydrolase; FBS: Fetal bovine serum; FCS: Fetal calf serum; MAGL: monoacylglycerol lipase: MS: multiple sclerosis; NAPE-PLD: N-acyl phosphatidylethanolamine phospholipase D; NGS: normal goat serum; PFU: plaque forming units; PPARs: peroxisome proliferator-activating receptors; RT: room temperature; SR1: SR141716A; TMEV: Theiler's murine encephalomyelitis virus; TMEV-IDD: TMEV-induced demyelinating disease; TRPV1: transient receptor potential cation channel: subfamily V: member 1; VCAM-1: vascular cell adhesion molecule-1; VLA-4: very late antigen-4.

## Competing interests

The authors declare that they have no competing interests.

## Authors' contributions

LM performed the majority of all experiments, participated in the design of the study, participated in the statistical analysis and drafted the manuscript. PMI participated in the adhesion experiments and revising manuscript draft. MM helped to immunohistochemistry studies and participated in the interpretation of data and revising manuscript draft. FC, FL and FD participated in the design of the study, interpretation of data and revision of manuscript draft. MH helped to performed *in vitro *experiments. JB participated in the design of the study, in the statistical analysis and revising manuscript draft. CG conceived of the study, and participated in its design and coordination and helped to draft the manuscript. All authors read and approved the final manuscript.

## Supplementary Material

Additional file 1 **Schematic drawing of the *in vitro *BBB model performance and experimental design**. Schematic drawing of the BBB model, experimental design and confirmation of BBB characteristic by Evan's blue permeability assay and Zonula occludens 1 immunocytochemistry. Click here for file

Additional file 2 **Schematic drawing of the *in vitro *BBB model performance and experimental design**. Schematic drawing of the BBB model, experimental design and confirmation of BBB characteristic by Evan's blue permeability assay and Zonula occludens 1 immunocytochemistry. Click here for file
