# Supplementary material for: Anandamide inhibits Theiler's virus induced VCAM-1 in brain endothelial cells and reduces leukocyte transmigration in a model of blood brain barrier by activation of CB1 receptors
Source: J Neuroinflammation. 2011 Aug 18;8:102. doi: 10.1186/1742-2094-8-102 (PMC3173342; doi:10.1186/1742-2094-8-102)
Supplement: Additional file 1 — Schematic drawing of the in vitro BBB model performance and experimental design. Schematic drawing of the BBB model, experimental design and confirmation of BBB characteristic by Evan's blue permeability assay and Zonula occludens 1 immunocytochemistry. [file 1742-2094-8-102-S1.PPT]

## Slide 1
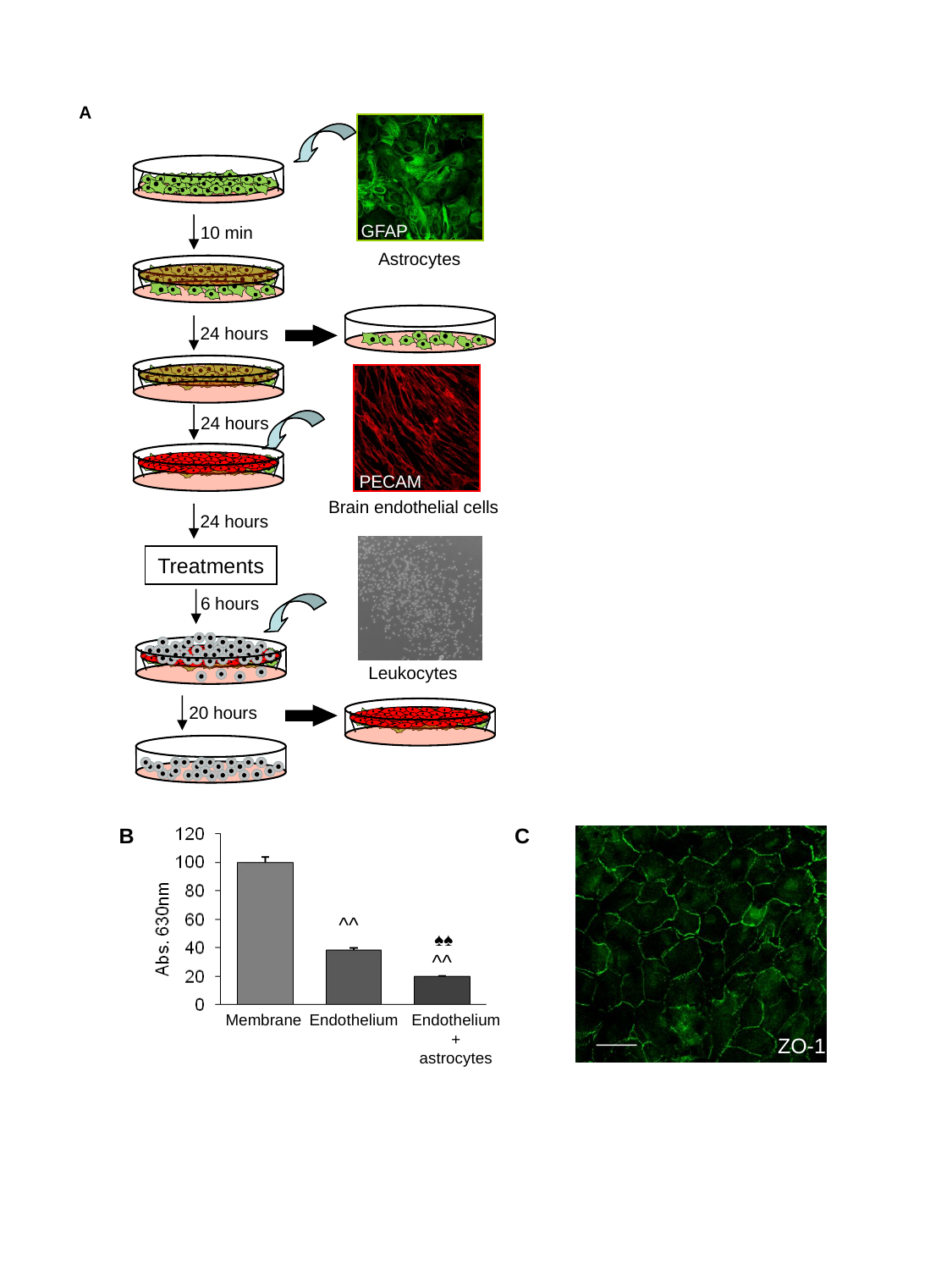

A
GFAP
10 min
Astrocytes
24 hours
PECAM
24 hours
Brain endothelial cells
24 hours
Treatments
6 hours
Leukocytes
20 hours
B
C
^^
♠♠
^^
Membrane
Endothelium
Endothelium +
astrocytes
ZO-1
