# Supplementary material for: Anandamide inhibits Theiler's virus induced VCAM-1 in brain endothelial cells and reduces leukocyte transmigration in a model of blood brain barrier by activation of CB1 receptors
Source: J Neuroinflammation. 2011 Aug 18;8:102. doi: 10.1186/1742-2094-8-102 (PMC3173342; doi:10.1186/1742-2094-8-102)
Supplement: Additional file 2 — Schematic drawing of the in vitro BBB model performance and experimental design. Schematic drawing of the BBB model, experimental design and confirmation of BBB characteristic by Evan's blue permeability assay and Zonula occludens 1 immunocytochemistry. [file 1742-2094-8-102-S2.DOC]

Additional file 1. Schematic drawing of the *in vitro* BBB model performance and experimental design. Membrane 8μm pore were coated with collagen type I and fibronectin (**A**) Astrocytes seeded on the bottom of the filter at a density of 5x10^5^ cells per filter were allowed to adhere for 10 min. After flip the insert and maintain the culture for 24 hours, the insert was changed to other well to avoid possible astrocytes attached to the bottom of the well contamination. 24 hours later, 5x10^5^ cells brain endothelial cells (b.End5) were seeded on the top of the filter membrane and allowed growing for 24 hours more. Treatments were added and culture was maintained for 6 hours. Leukocytes at a density of 2.5x10^5^ were added to the upper side of the insert for 20 hours. Insert was removed and leukocytes in the bottom of the well were photographed and counted by using hemocytometer. (**B**) Confirmation of BBB characteristic. Absorbance at 630 nm was measured to analyze the permeability to 0.45% albumin conjugated with evan’s blue to cross a membrane, a endothelial monolayer or the BBB model. (^^p<0.01 vs. Membrane; ♠♠p<0.01 vs. endothelium, ANOVA followed Tuckey’s test). (**C**) Representative immunofluorescence micrograph of brain endothelial cells and astrocytes cultured on an insert stained for the tight junction ZO-1. Scale bar 50 μm.
